# Tyrosine Kinase-Dependent Defense Responses Against Herbivory in Arabidopsis

**DOI:** 10.3389/fpls.2019.00776

**Published:** 2019-06-12

**Authors:** Takumi Miyamoto, Takuya Uemura, Keiichirou Nemoto, Maho Daito, Akira Nozawa, Tatsuya Sawasaki, Gen-ichiro Arimura

**Affiliations:** ^1^Department of Biological Science and Technology, Faculty of Industrial Science and Technology, Tokyo University of Science, Tokyo, Japan; ^2^Iwate Biotechnology Research Center, Iwate, Japan; ^3^Proteo-Science Center, Ehime University, Matsuyama, Japan

**Keywords:** *Arabidopsis thaliana*, calcium-dependent protein kinase related protein kinase (CRK), defense response, *Spodoptera litura*, tyrosine kinase

## Abstract

Tyrosine (Tyr) phosphorylation (TP) is important for promotion of plants’ signaling. Arabidopsis calcium-dependent protein kinase related protein kinases (CRK2 and CRK3) phosphorylate Tyr residues of a subset of transcription factors (TFs), including herbivory-responsive ethylene response factor 13 (ERF13), but the *in vivo* functions of these kinases in plant defense responses and development remain to be clarified. We show that when CRKs were coexpressed with ERF13 in Arabidopsis leaf protoplasts, the transcription activity regulated via ERF13 was elevated by CRK2 but not CRK3 or their kinase-dead form mutants. Moreover, this elevation was abolished when a Tyr-phosphorylation mutant of ERF was coexpressed with CRK2, indicating that CRK2 serves as an effector of ERF13 mediated by Tyr-phosphorylation. Moreover, CRK2 and CRK3 acted as effectors of RAP2.6 and WRKY14, respectively. CRK-overexpressing lines and knockout mutants of Arabidopsis plants showed increased and decreased expression levels of the defensin gene *PDF1.2* in leaves, respectively, conferring on the plants modulated defense properties against the generalist herbivore *Spodoptera litura*. However, these lines did not show any obvious developmental defects, indicating that CRKs play a role in defense responses but not in the ordinary growth or development of plants. Transcription of both *CRK2* and *CRK3* was positively regulated by jasmonate signaling and abscisic acid (ABA) signaling upon herbivory. Our findings suggest that these phytohormone-responsive CRKs work coordinately for plant defense responses via Tyr phosphorylation of herbivory-responsive regulators.

## Introduction

Tyrosine (Tyr) phosphorylation (TP) is a notable regulator of signal transduction in eukaryotic cells (Blume-Jensen and Hunter, 2001). It has been estimated that in *Arabidopsis thaliana* (Arabidopsis) and rice, 4% of phosphopeptides are Tyr-phosphorylated peptides, which is similar to the proportion in humans (Nakagami et al., 2010). Given the fact that TP is involved in abscisic acid (ABA) signaling (Ghelis et al., 2008), gibberellin responses (Fu et al., 2002), cold stress (Sangwan et al., 2001), and sugar responses (Ritsema et al., 2009), TP is considered to be multiply involved in not only plant growth and development but also defense responses to biotic and abiotic stresses.

Notably, Nemoto et al. (2015) recently reported that Arabidopsis calcium-dependent protein kinase (CPK)-related protein kinases [CRK2 (At3g19100) and CRK3 (At2g46700)] phosphorylate Tyr residues of beta-tubulin and an array of transcription factors (TFs), including ethylene response factor 13 (ERF13) (At2g44840), WRKY DNA-binding protein 14 (WRKY14) (At1g30650), ERF subfamily B-4 member ERF/AP2 transcription factor 2.6 (RAP2.6) (At1g43160), and cryptochrome-interacting basic-helix-loop-helix 5 (CIB5) (At1g26260). The transcript level of ERF13 in Arabidopsis leaves is responsive to exogenous ABA and jasmonate (JA) application, suggesting that ERF13 is relevant to plant stress responses (Lee et al., 2010; Schweizer et al., 2013). RAP2.6, another member of the ERF family, has also been shown to function in plant defense responses to nematodes, ABA, salt and osmotic stresses (Zhu et al., 2010; Ali et al., 2013; Guo and Sun, 2017). The same holds true for the WRKY gene family, which plays key roles in plant stress responses, including toward biotic stress (Eulgem et al., 2000; Chen et al., 2012; Birkenbihl et al., 2018). Notably, in *Coptis japonica*, TP has been proposed to enhance the nuclear localization, DNA-binding activity and transactivation of a WRKY involved in regulating the biosynthesis of the defensive products benzylisoquinoline alkaloids (Yamada and Sato, 2016). It is therefore clear that TP plays central roles in cellular signaling of plant stress responses. CRK2 and CRK3 share 57.7% amino acid identity, and they share a serine (Ser)/threonine (Thr) kinase domain and a degenerate calcium-binding EF-hand motif. In spite of the structural similarity of CRKs to typical Ser/Thr-type protein kinases, CRK2 and CRK3 preferentially phosphorylate tyrosine residues in the absence of calcium (Nemoto et al., 2015).

On the other hand, Ser/Thr phosphorylation has been classically focused on regarding its relevance to plants’ stress responses. For instance, it has been elucidated that CPK2 (NtCDPK2) modulates the activation level of stress-induced mitogen-activated protein kinases (MAPKs), leading to increased levels of the defense-associated phytohormones JA, 12-oxo-phytodienoic acid, and ethylene in tobacco (Ludwig et al., 2005). Moreover, Arabidopsis AtCPK3 and AtCPK13 have been reported to activate a heat shock TF (HsfB2a) involved in activation of the defense gene *PDF1.2* in Arabidopsis plants infested by larvae of the generalist herbivore *Spodoptera litura* (Nagamangala Kanchiswamy et al., 2010). In contrast to these Ser/Thr kinases, however, the nature of Tyr kinases that act in plant defense responses remains obscure. We therefore focused on CRKs involved in the phosphorylation of defense-associated TFs. Here we show that CRK2 and CRK3 play a central role in eliciting defense responses of Arabidopsis host plants against the generalist herbivore *S. litura*. Moreover, since CRKs are also known to be involved in gibberellin signaling through the phosphorylation of GARU (gibberellin receptor RING E3 ubiquitin ligase), leading to ubiquitin-dependent degradation of gibberellin receptor (GID1) in Arabidopsis seedlings (Nemoto et al., 2017), phenotypic analyses were carried out using CRK overexpression and mutant lines.

## Materials and Methods

### Plants

Wild-type (WT) Arabidopsis ecotype Col-0 plants, CRK T-DNA insertion mutants [*crk2* [Salk_090938C], and *crk3* [Salk_128719C] (Nemoto et al., 2015)], ABA INSENSITIVE 1 mutant [*abi1-1* (Allen et al., 1999)], ETHYLENE INSENSITIVE 2 mutant [*ein2* (Solano et al., 1998)], and transgenic plants overexpressing *CRK2* or *CRK3* (see below) were grown in plastic pots for 4–5 weeks in a growth chamber at 22 ± 1°C with a photoperiod of 14 h (80 μE m^−2^ s^−1^). WT of Arabidopsis ecotype Landsberg erecta (Ler) and its *erf13* mutant (CS26912) were grown in these same growth conditions. The CORONATINE INSENSITIVE1 (COI1) mutant (*coi1-1*; Col-0 background) seeds were germinated on 1/2 Murashige and Skoog (MS) medium supplemented with 2% sucrose, 0.8% agar, and 50 μM methyl jasmonate (MeJA, Wako Pure Chemical Industrials, Ltd., Osaka, Japan) to screen the individuals showing normal root growth for 2 weeks (Xie et al., 1998). The screened plants were transferred and grown in plastic pots for an additional 3 weeks.

### Chemical and Herbivore Treatments

*S. litura* were reared on an artificial diet (Insecta LF, Nihon Nosan Nogyo Ltd., Tokyo, Japan) in the laboratory at 24 ± 1°C with a photoperiod of 16 h. For herbivore treatment, four third-instar larvae per plant were released on potted Arabidopsis plants in a growth chamber for 24 h. After chemical and herbivore treatment, all the plants were incubated at 22 ± 1°C (14 h photoperiod at a light intensity of 80 μE m^−2^ s^−1^).

Arabidopsis plants were evenly sprayed with 1 mL of aqueous solutions (0.1% (v/v) ethanol) of MeJA (Wako Pure Chemical industrials; 0.2 mM), 1-aminocyclopropane-1-carboxylic acid (ACC, Wako Pure Chemical Industries; 0.01 mM) or ABA (Tokyo Chemical Industry Co., Ltd., Tokyo, Japan; 0.1 mM) and incubated in a growth chamber for up to 24 h.

### Primers

Primers used for all the polymerase chain reactions (PCRs) in this study are listed in Supplementary Table S1.

### Protoplast Preparation and Transfection

**FIGURE 1:**
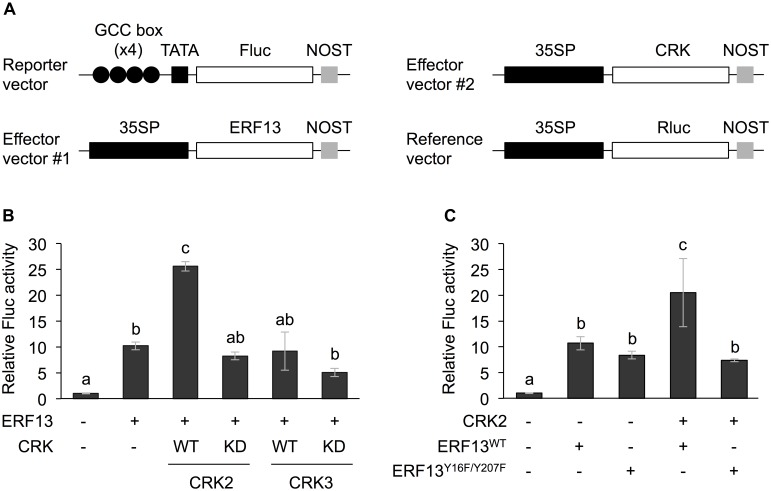
Dual luciferase (LUC) activity mediated through CRK-activated ERF13. **(A)** Schematic diagram of the reporter and effector vectors used in dual LUC assays. **(B)** Four inverted repeats of GCC-box fused to a minimal TATA-box and a firefly LUC (Fluc) reporter gene. Transient activation of the reporter gene according to co-expression with (+) or without (-) ERF13, wild-type (WT) and kinase dead-mutant (KD) of CRK2 or CRK3 in Arabidopsis protoplasts was assessed. **(C)** Likewise, transient activation of the reporter gene according to co-expressed effector(s), CRK2, WT of ERF13 (ERF13^WT^), or ERF13 mutant deficient in CRK-phosphorylated sites (ERF13^Y 16F/Y 207F^) in Arabidopsis protoplast cells was assessed. Data represent the mean and standard error (*n* = 3). Renilla luciferase (Rluc) activity was used to normalize for the efficiency of transformation. Means indicated by different small letters are significantly different, based on a one-way ANOVA with *post hoc* Tukey’s HSD (*P* < 0.05). NOST, nopaline synthase terminator; 35SP, cauliflower mosaic virus 35S promoter; and TATA, TATA-box.

The full-length coding region of *CRK2*, *CRK3*, or *ERF13* was cloned into the p35SΩ-GW-NOST vector [cauliflower mosaic virus 35S promoter (35SP)::Ω sequence (translation enhancer)::the Gateway cassette (GW) region::Nopaline synthase terminator (NOST) (Nemoto et al., 2015)] using the Gateway cloning system (Thermo Fisher Scientific, Waltham, MA, United States). The kinase dead (*KD*) form mutants of CRK2 (Lys_176_ to Arg [CRK2^KD^]) and CRK3 (Lys_175_ to Arg [CRK3^KD^]), and ERF13 mutants (Tyr_16_ to Phe and Tyr_207_ to Phe [ERF13^Y 16F/Y 207F^]) were generated using a PrimeSTAR Mutagenesis Basal Kit (Takara Bio Inc., Otsu, Japan) according to the manufacturer’s instructions (Nemoto et al., 2015). These cDNAs were also inserted into the GW region of p35SΩ-GW-NOST vector. Four repeat sequence of a GCC-box (AGCCGCC) fragment or a W-box (TTTGACC) fragment was fused to a minimal TATA box::a firefly luciferase (Fluc) reporter gene::NOST in the pMA cloning vector (Thermo Fisher Scientific, Waltham, MA, United States). The map of the representative vectors used is shown in Figure 1.

Protoplast isolation from Arabidopsis leaves was performed as previously described (Wu et al., 2009). The peeled leaves (4-5-week-old plants), still adhering to the tape, were transferred to a Petri dish containing 10 ml of enzyme solution (2% (w/v) cellulase “Onozuka” R10 [Yakult Pharmaceutical Industry, Tokyo, Japan], 0.3% (w/v) macerozyme “Onozuka” R10 [Yakult Pharmaceutical Industry], 0.4 M mannitol, 10 mM CaCl_2_, and 5 mM MES [pH 5.7]). The leaf tissues were incubated at room temperature for 1 h until the protoplasts were sufficiently released into the solution. The protoplasts isolated were diluted with an equal volume of W5 solution (154 mM NaCl, 125 mM CaCl_2_, 5 mM KCl, and 2 mM MES [pH 5.7]) and filtered with filter paper to remove undigested leaf tissues. The protoplast suspension was centrifuged at 100 *g* for 2 min and re-suspended with W5 solution to adjust it to 2 × 10^5^ cells ml^−1^. The protoplast suspension was centrifuged again and finally resuspended in an equal volume of modified MMG solution (0.4 M mannitol, 15 mM MgCl_2_, and 5 mM MES [pH 5.7]).

Polyethylene glycol-mediated DNA transfection was performed as previously described (Yoo et al., 2007). The protoplast suspension (100 μl) was supplemented with a mixture of vectors carrying 35SP::CRK (CRK2^WT^, CRK2^KD^, CRK3^WT^, or CRK3^KD^)::NOST, 35SP::TF (ERF13^WT^, ERF13^Y 16F/Y 207F^, WRKY14, or RAP2.6)::NOST, GCC-box or W-box::TATA::Fluc::NOST and reference (35SP::Renilla luciferase [Rluc]::NOST) vector at a ratio of 4:5:5:1 to protoplast suspension with 110 μl PEG solution [40% (w/v) polyethylene glycerol, 0.4 M mannitol, and 0.1 M Ca(NO_3_)_2_4H_2_O]. The transfection was carried out at room temperature for 5 min and stopped by adding 400 μl of W5 solution. The protoplasts were collected by centrifugation at 100 *g* for 2 min and resuspended with 500 μl of WI solution (5 mM MES [pH 5.7], 0.4 M mannitol, and 20 mM KCl) and incubated in a 12-well tissue culture plate at room temperature overnight.

### Luciferase (LUC) Assay

The LUC assay was performed as previously described (Luehrsen et al., 1992). The protoplasts were collected by centrifugation at 100 *g* for 2 min, and re-suspended with 100 μl of EX buffer (50 mM Tris–HCl [pH 8.0], 150 mM NaCl, and 0.5% (v/v) Triton X-100). The protoplasts were again centrifuged at 20,000 *g* for 10 min at 4°C, and 10 μl of supernatant was used for a LUC assay. LUC activity was measured with a 1420 Luminescence Counter ARVO Light (Perkin Elmer, Waltham, MA, United States) using the Dual-Luciferase^®^Reporter assay system (Promega, Madison, WI, United States). Fluc activity produced due to the transfected reporter construct was expressed as the value normalized by the Rluc activity produced due to the co-transfected reference vector. Replicate analyses were conducted with 3 independent samples.

### Generation of Transgenic Arabidopsis Plants

The full-length coding region of *CRK2* or *CRK3* was inserted into binary vector pMDC32 (2x 35SP::GW::NOST) using the Gateway cloning system (see above). The resulting vector, pMDC32-*CRK2*, pMDC32-*CRK3* or pMDC32 [vector control (VC)], was transformed into *Agrobacterium tumefaciens* strain EHA105 by electroporation. WT Arabidopsis plants that had been grown for about 6–7 weeks were transformed via the floral-dip transformation method (Clough and Bent, 1998). Transgenic T1 seeds from each transformant were tested for germination on 1/2 MS medium supplemented with 30 mg l^−1^ hygromycin. T2 seeds harvested from each individual T0 plant that showed ca. 3:1 segregation ratio was tested for hygromycin-resistance again. T3 homozygous plant lines were used for further analyses.

### RNA Extraction, cDNA Synthesis and Quantitative PCR (qPCR)

RNA extraction, first-strand cDNA synthesis and quantitative PCR were performed according to the method described previously (Ali et al., 2019).

### Herbivore Assay

We performed assays to assess the growth of *S. litura* larvae at 22 ± 1°C (14 h photoperiod at 80 μE m^−2^ s^−1^). Third-instar larvae were initially weighed (1.7–2.1 mg), and each larva was released onto a potted plant for 3 days. The net body weight that *S. litura* larvae gained each of the following 3 days was determined. When a larva died or was lost during the assay, we excluded that sample, and final replicate analyses were conducted with 16–22 independent samples.

### Root Length Measurement

Plant seedlings (14 days old) were grown on 1/2 MS medium. Root lengths were determined using ImageJ software [version 1.50i; (Schneider et al., 2012)].

### Statistical Analysis

We performed *t*-tests for pairwise analysis and one-way ANOVA with Holm’s sequential Bonferroni *post hoc* test or Tukey’s HSD test using the program^1^ for comparing multiple samples.

## Results

### *In vivo* Function of CRK2 in Transactivation of ERF13

Both CRK2 and CRK3 phosphorylate ERF13 at two Tyr residues (Y16 and Y207) (Nemoto et al., 2015). To investigate the roles of CRK-promoted TP in ERF13 transactivation, CRKs were expressed together with ERF13 as an activator of a reporter (firefly LUC [Fluc]) gene coexpressed under the control of a chimeric promoter that consisted of four inverted repeats of GCC-box [ERF-binding *cis*-element (Fujimoto et al., 2000)] fused to a minimal TATA-box, in Arabidopsis mesophyll protoplasts (Figure 1A). Expression of ERF13 caused a 10-fold increase of Fluc activity in comparison to the activity in the absence of ERF13 (Figure 1B). When WT CRK2 (CRK2^WT^) was coexpressed with ERF13, an additional 2.5-fold increase of Fluc activity was detected. However, transactivation of ERF13 was not caused by either CRK3 or kinase domain-mutant KD CRK2 (CRK2^KD^, whose lack of kinase activity has been shown previously; Nemoto et al., 2015, 2017) when they were concomitantly expressed in the cells. Moreover, when an ERF13 mutant deficient in CRK-phosphorylated sites (ERF13^Y 16F/Y 207F^), instead of WT ERF13 (ERF13^WT^), was co-expressed with CRK2, the Fluc activity declined to the basal level achieved by the expression of ERF13^WT^ alone (Figure 1C).

### Transactivation of WRKY14 and RAP2.6 by CRK

CRK2 and CRK3 are also able to phosphorylate WRKY14 (Nemoto et al., 2015), one of the WRKY members involved in an array of plant defense responses (Skibbe et al., 2008; Bakshi and Oelmuller, 2014; Li et al., 2015). Moreover, RAP2.6, another AP2/ERF protein member involved in plant stress responses (Krishnaswamy et al., 2011; Ali et al., 2013), has been shown to be phosphorylated by CRK2 but not CRK3 (Nemoto et al., 2015). We therefore explored those two TFs as substrate targets for CRKs, utilizing the transient Fluc expression system in protoplast cells, using the two *cis*-elements, i.e., a W-box and a GCC-box for WRKY14 (Chen et al., 2012) and RAP2.6 (Zhu et al., 2010), respectively.

**FIGURE 2:**
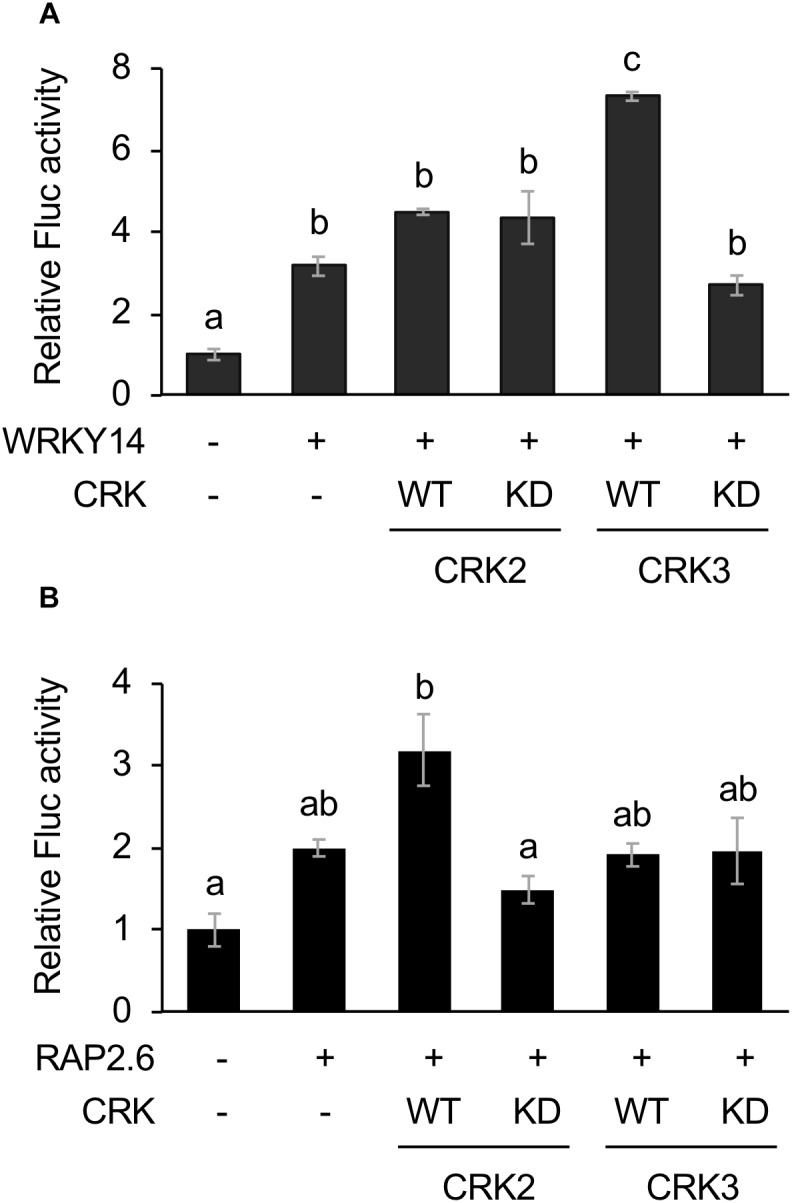
Dual luciferase (LUC) activity mediated through CRK-activated WRKY14 or RAP2.6. Four inverted repeats of W-box or GCC-box fragment fused to a minimal TATA-box and a firefly LUC (Fluc) reporter gene. Transient activation of the reporter gene according to co-expression with (+) or without (-) WRKY14 **(A)** or RAP2.6 **(B)**, WT or kinase dead-mutant (KD) of CRK2 or CRK3 in Arabidopsis protoplast cells was assessed. Renilla luciferase (Rluc) activity was used to normalize for the efficiency of transformation. Data represent the mean and standard error (*n* = 3). Means indicated by different small letters are significantly different, based on a one-way ANOVA with *post hoc* Tukey’s HSD (*P* < 0.05).

The Fluc activity was increased by WRKY14 expression. This activity marginally tended to be elevated by coexpression of WT CRK3 (CRK3^WT^) (Figure 2A). However, this transactivation was achieved by neither CRK3^KD^ nor CRK2 (CRK2^WT^ or CRK2^KD^). In contrast, Fluc activity, which was only marginally increased by expression of RAP2.6, was elevated by coexpression of CRK2^WT^ (Figure 2B). Again, this transactivation was not achieved by coexpression of CRK2^KD^ or CRK3 (CRK3^WT^ or CRK3^KD^).

### Defense Ability and Growth/Development of CRK Mutants and Overexpressing Lines

We obtained two lines and three lines of CRK2- or CRK3-overexpressing plants, respectively. Two respective representative lines (CRK2-OX2 and CRK3-OX3) exhibited 170-fold and 70-fold increased levels of *CRK2* and *CRK3* expression under the constitutive 35SP, respectively, compared to the levels in leaves of the VC line (Supplementary Figure S1).

**FIGURE 3:**
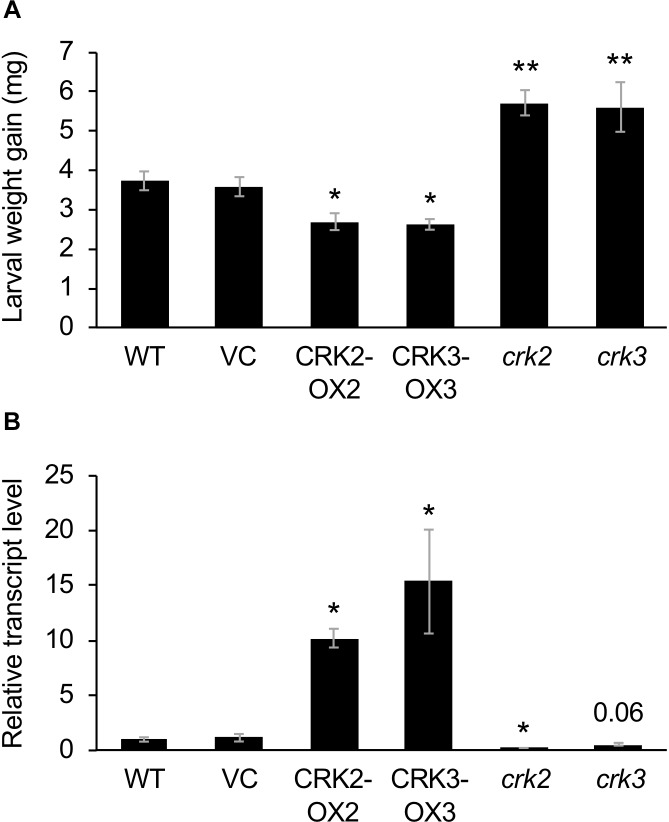
Defense property of wild type (WT), *crk* mutants, and CRK-overexpressing lines. **(A)** The net body weight that *Spodoptera litura* larvae gained during 3 days after they had been placed on potted plants of WT, *crk2*, or *crk3* mutants, CRK-overexpressing lines (CRK2-OX2 and CRK3-OX3) and their vector control (VC) lines. Data represent the mean and standard error (*n* = 16–22). **(B)** Transcript levels of a defensin gene *PDF1.2* in the leaves of WT, *crk* mutants, CRK-overexpressing plants, and VC plants. Transcript levels of genes were measured by RT-qPCR and normalized by those of *ACT8*. Data represent the mean and standard error (*n* = 4 for VC, CRK2-OX2, and CRK3-OX3; *n* = 7 for WT, *crk2*, and *crk3*). Data marked with an asterisk are significantly different from those of WT **(A)** or VC **(B)**, based on a one-way ANOVA with Holm’s sequential Bonferroni *post hoc* test (^∗∗^*P* < 0.01, ^∗^0.01 ≤*P* < 0.05). Otherwise, the mean followed by *P* value is marginally different from the control value.

These transgenic lines exhibited lower development of larvae of the generalist herbivore *S. litura* hosted on the potted plants for 3 days, compared to that on VC plants (Figure 3A). This was in agreement with the constitutively elevated expression levels of the JA-inducible plant defensin gene *PDF1.2* (Manners et al., 1998) in leaves of these two transgenic lines (Figure 3B). In contrast, *crk2* and *crk3* knockdown mutants (Nemoto et al., 2015) exhibited enhanced development of larvae on the potted plants during 3 days, in accord with the constitutively lower expression level of *PDF1.2* in their leaves (Figure 3A,B). The GCC-box located at −255 to −261 in the *PDF1.2* promoter has been shown to play a key role in conferring JA responsiveness to *PDF1.2* expression (Brown et al., 2003). Putative W-boxes (TGACC/T) are also located at −388 to −384, −773 to −768, and −828 to −823 in the *PDF1.2* promoter upstream region.

**FIGURE 4:**
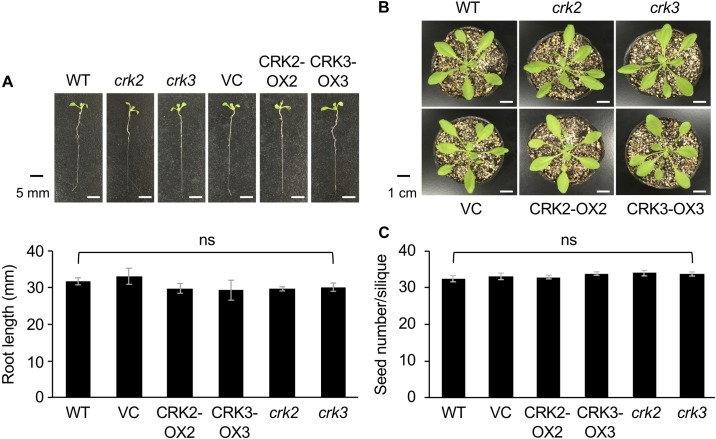
The phenotype and root length of seedlings **(A)** and rosette plants **(B)**, and the numbers of seeds **(C)** of wild type (WT), *crk2*, and *crk3* mutants, CRK-overexpressing lines (CRK2-OX2 and CRK3-OX3), and their VC line. Plant seedlings were grown on medium for 14 days, and rosette plants and plants during the harvest time were grown on soil for 4 weeks and ∼8 weeks, respectively. Means and standard errors of root lengths were determined using 12 individual seedlings. Means and standard errors of the numbers of seeds were determined using five pods from eight individual plants. ns, not significant based on a one-way ANOVA.

None of the transgenic lines or mutants of CRKs showed any marked differences in plant growth, development or morphology, including root growth, vegetative stage development, or seed number (Figure 4).

### Transcriptional Regulation of *CRK2* and *CRK3* via Phytohormone Signaling for Defense Responses

**FIGURE 5:**
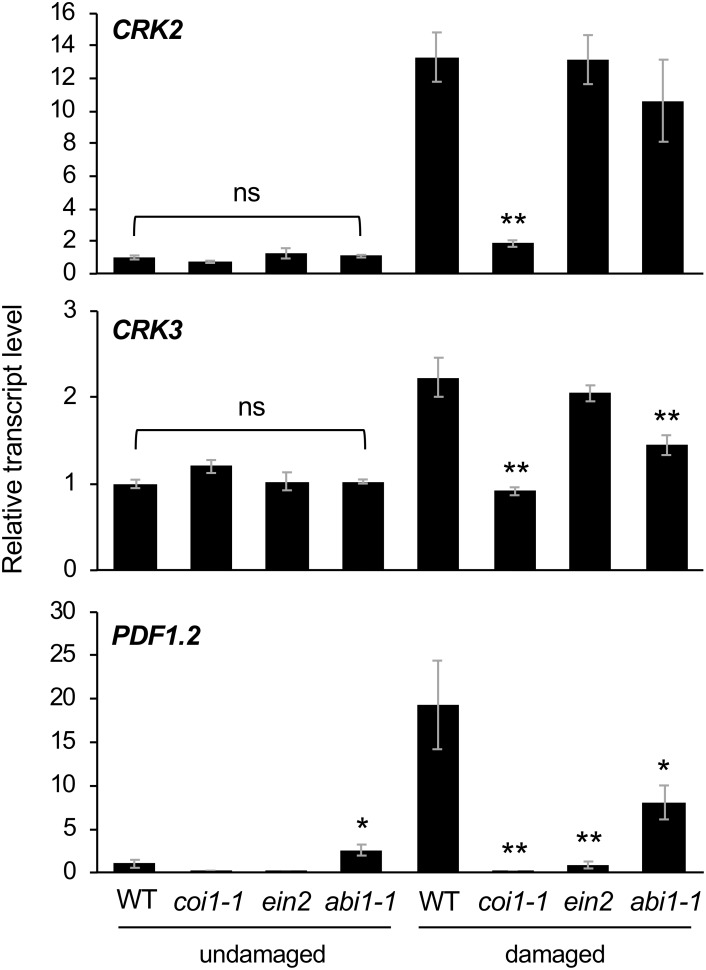
Transcriptional regulation of *CRKs* in infested leaves. Transcript levels of *CRK2*, *CRK3*, and *PDF1.2* in leaves of Arabidopsis wild-type (WT), *coi1-1*, *abi1-1*, and *ein2* plants damaged or not with *S. litura* larvae for 24 h. Transcript levels of genes were measured by RT-qPCR and normalized by those of *ACT8*. Data represent the mean and standard error (*n* = 5–8). Data marked with an asterisk are significantly different from those of undamaged or damaged WT plants, based on a one-way ANOVA with Holm’s sequential Bonferroni *post hoc* test (^∗∗^0.001 ≤*P* < 0.01, ^∗^0.01 ≤*P* < 0.05). ns, not significant.

Finally, Arabidopsis mutant plants defective in JA signaling (*coi1-1*) (Xie et al., 1998), ethylene signaling (*ein2*) (Ju and Chang, 2015), and ABA signaling (*abi1-1*) (Pei et al., 1997) were assessed to evaluate the involvement of hormone signaling in *CRK* activation during damage by *S. litura* (Figure 5). In comparison to the induction of transcripts of *CRK2* and *CRK3* in WT leaves, *coi1-1* plants exhibited defective elevation of these transcript levels in leaves upon herbivory, as did *PDF1.2* plants. *ein2* and *abi1-1* plants did not show defective elevation of transcript levels of *CRK2* upon herbivory. However, *abi1-1* plants showed defective elevation of the *CRK3* transcript level upon herbivory. Although *abi1-1* leaves showed slightly higher expression of *PDF1.2* compared to WT in undamaged leaves, this issue was not further explored because it was outside the focus of the present study.

**FIGURE 6:**
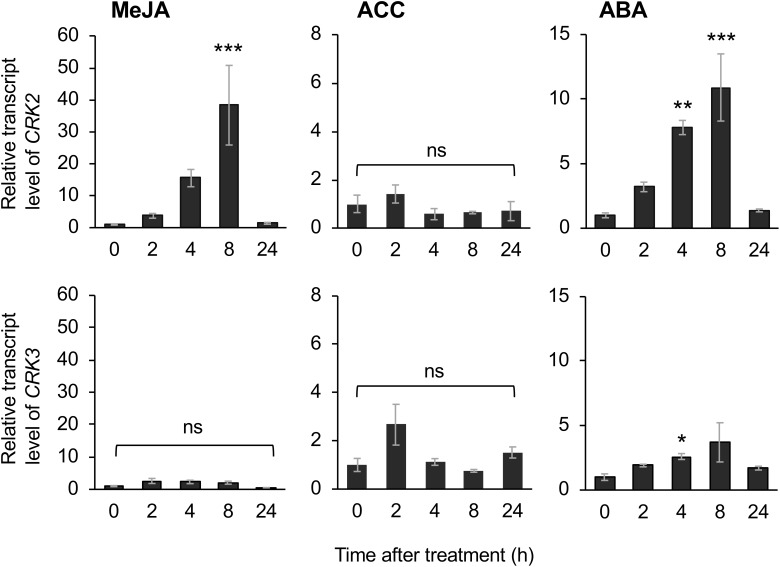
Phytohormone-induced regulation of CRK expression. Transcript levels of *CRK2* and *CRK3* in leaves of Arabidopsis wild-type plants in response to exogenous application of methyl jasmonate (MeJA), an ethylene precursor [1-aminocyclopropane-1-carboxylic acid (ACC)], abscisic acid (ABA) for up to 24 h. Transcript levels of genes were measured by RT-qPCR and normalized by those of *ACT8*. Data represent the mean and standard error (*n* = 6). Data marked with an asterisk are significantly different from those of undamaged or damaged WT plants, based on a one-way ANOVA with Holm’s sequential Bonferroni *post hoc* test (^∗∗∗^*P* < 0.001, ^∗∗^0.001 ≤*P* < 0.01; ^∗^0.01 ≤*P* < 0.05). ns, not significant.

The application of exogenous phytohormone solutions to WT plants resulted in induced expression of *CRK2* in leaves treated with the methyl form of JA (MeJA) or ABA but not an ethylene precursor (ACC) (Figure 6). Moreover, expression of *CRK3* was elicited at 4 h in leaves treated with ABA but not MeJA or ACC for up to 24 h.

## Discussion

Tyr phosphorylation mediated by Arabidopsis CRKs appeared to modulate the activities of TFs including ERF13, WRKY14, and RAP2.6 (Figure 1, 2). Although ERF13 and WRKY14 were previously shown to be phosphorylated by both CRK2 and CRK3 using an *in vitro* phosphorylation system (Nemoto et al., 2015), the transactivation of ERF13 and WRKY14 was achieved by CRK2 and CRK3 alone, respectively (Figure 1, 2). However, these findings are not surprising because *in vitro* phosphorylation activity does not always accord with the *in vivo* functions (Delom and Chevet, 2006). This may be because the concentrated kinase protein and/or possibly contaminating kinases from the eukaryotic protein synthesis system cause non-specific phosphorylation of substrate targets in *in vitro* assays. Moreover, it is known that phosphorylation modification of TFs can be responsible not only for their transactivation but also for their nuclear translocation (Liu et al., 2017) as well as enhancement of their binding to the particular *cis*-element of the respective promoter region (Gao et al., 2013).

**FIGURE 7:**
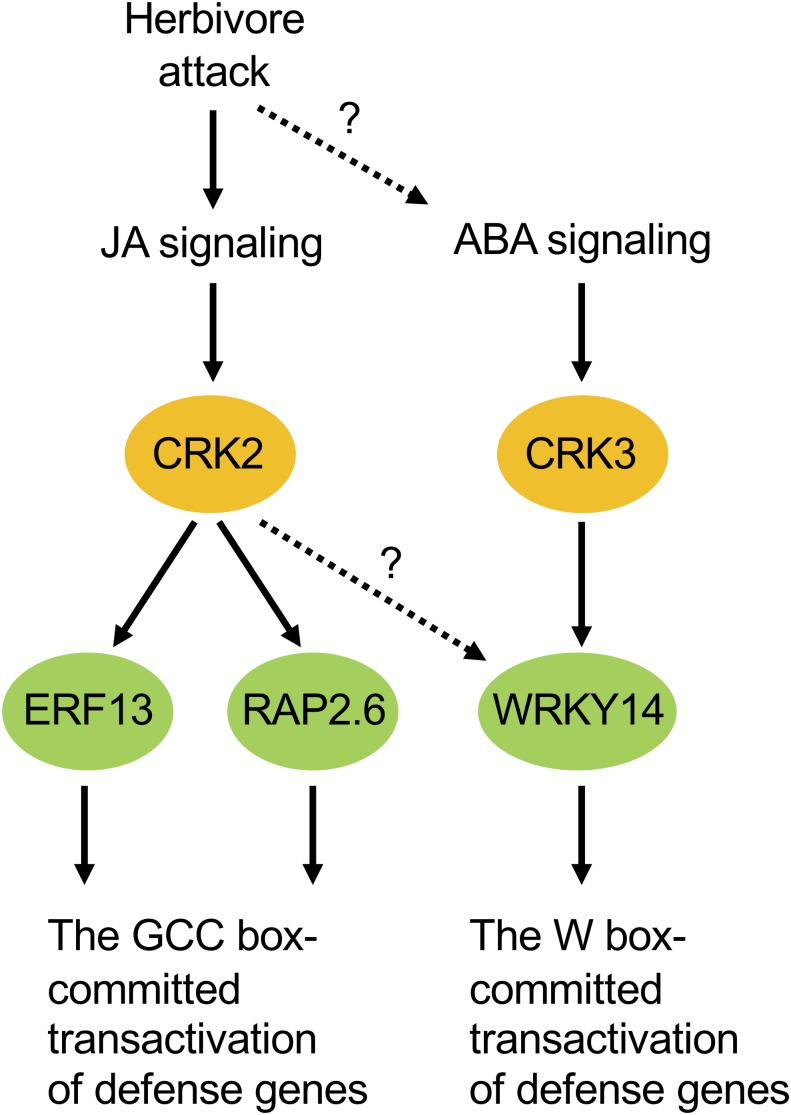
Possible model of cellular signaling mediated by CRKs under herbivore attack. ABA, abscisic acid; JA, jasmonate.

According to our phenotypic characterization of loss and gain of CRK functions, it appeared that both CRK2 and CRK3 are involved in plant defense responses to *S. litura* damage (Figure 3). However, it is important to note that the *erf13* mutant did not fully modulate the herbivore performance or the *PDF1.2* transcript in leaves, compared to those in WT (Supplementary Figure S2), in accord with previous findings (Schweizer et al., 2013). All these facts lead us to propose a model in which multiple CRK substrates, including not only ERF13 but also WRKY14, RAP2.6 and unknown TF substrates, may individually and/or synergistically coordinate the upregulation of defense genes such as *PDF1.2* (Figure 7). In addition, CRKs may control various regulatory molecules besides TFs in cellular signaling. For example, CRK2 phosphorylates GARU, a protein involved in ubiquitin-dependent degradation of the gibberellin receptor GID1 in gibberellin signaling of Arabidopsis seedlings (Nemoto et al., 2017). Both *garu* mutant and CRK2-OX plants enhance GID1 stabilization and DELLA degradation, indicating that CRK2 is positively involved in gibberellin signaling through the CRK2-mediated Tyr phosphorylation of GARU in Arabidopsis seedlings (Nemoto et al., 2017).

Moreover, ABA-responsive CRK3 (Figure 5) may be involved in *in planta* responses to not only herbivory but also pathogenesis and multiple environmental stresses. For example, WRKY, a substrate of CRK3, should especially function in gene regulation for environmental stress tolerance (Chen et al., 2012), and TP has been shown to be involved in ABA signaling (Ghelis et al., 2008), as described above. On the other hand, given the transcriptional profile of *CRK2* in the leaves of MeJA-treated WT plants and infested *coi1-1* plants, there is no doubt that JA is a master switch for *CRK2* activation in the infested leaves (Figure 5, 6, 7). In contrast to this, although ABA application activates *CRK2* expression, ABA is not likely to contribute to herbivory-response signaling, considering the data observed using *abi1-1*. In contrast, *CRK3* expression was not responsive to JA, but JA is likely involved in herbivory-response signaling according to data observed using *coi1-1*. Regarding this, we presume that *CRK3* is not directly activated by JA signaling, but probably concomitant effects from other signaling pathways such as ABA signaling, in concert with defense-signaling cross-talk (Erb et al., 2012), might affect *CRK3* expression in *coi1-1* leaves during herbivory.

Finally, it should be remarked that neither CRK-OX nor mutant lines show any phenotypic defects in plant growth, development or morphology in comparison to WT plants (Figure 4). Thus, CRKs are not likely to be relevant to plant development in the normal growth condition, although this seems to be paradoxical to the above-described possible involvement of CRK2 in gibberellin signaling. We therefore propose a possible model that CRK2 does not play a significant role in the GID1/GARU system under the normal condition, in which a low threshold level of endogenous gibberellin is maintained. However, when plants suffer from threats such as a lack of nutrients or biotic/abiotic stresses, plants switch to reduced endogenous gibberellin levels (Wild et al., 2012; Colebrook et al., 2014), and then CRK2 is recruited to play a primary role in gibberellin signaling. In other words, CRKs do serve under certain conditions for plants’ defense responses to environmental threats and plant growth/development, mediated through the assistance of phytohormone signaling.

## Data Availability

The datasets for this manuscript are not publicly available because data has not yet been linked to any public still domains. Requests to access the datasets should be directed to G-iA, garimura@rs.tus.ac.jp.

## Author Contributions

TM, TU, KN, MD, TS, and G-iA contributed to the conception and design the study. TM, TU, KN, MD, and AN performed the experiments. G-iA wrote the first draft of the manuscript. TM, TU, KN, and G-iA wrote sections of the manuscript.

## Conflict of Interest Statement

The authors declare that the research was conducted in the absence of any commercial or financial relationships that could be construed as a potential conflict of interest.

## Footnotes

1 http://astatsa.com/OneWay_Anova_with_TukeyHSD/
