# Supplementary material for: Tyrosine Kinase-Dependent Defense Responses Against Herbivory in Arabidopsis
Source: Front Plant Sci. 2019 Jun 12;10:776. doi: 10.3389/fpls.2019.00776 (PMC6582402; doi:10.3389/fpls.2019.00776)
Supplement: Supplementary file 1 [file Data_Sheet_1.PDF]

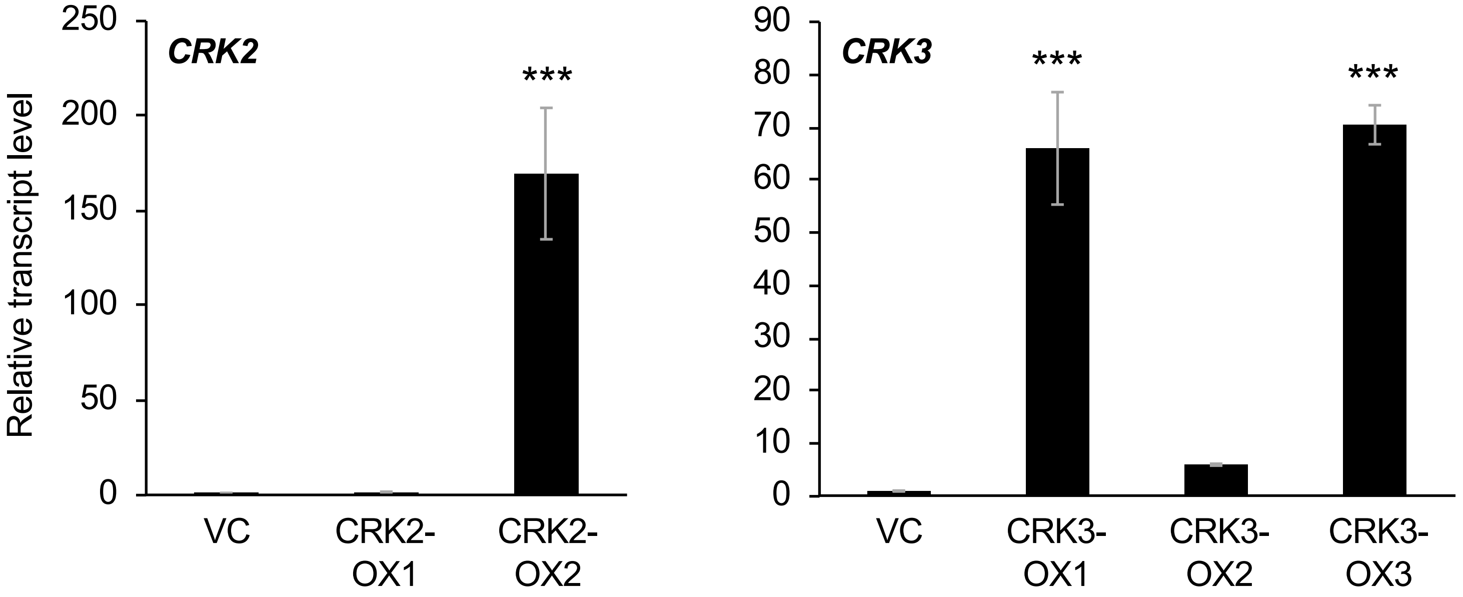
CRK2
***
250
200
150
100
50
0
Relative transcript level
VC
CRK2-OX1
CRK2-OX2
CRK3
***
***
90
80
70
60
50
40
30
20
10
0
VC
CRK3-OX1
CRK3-OX2
CRK3-OX3

Figure S1

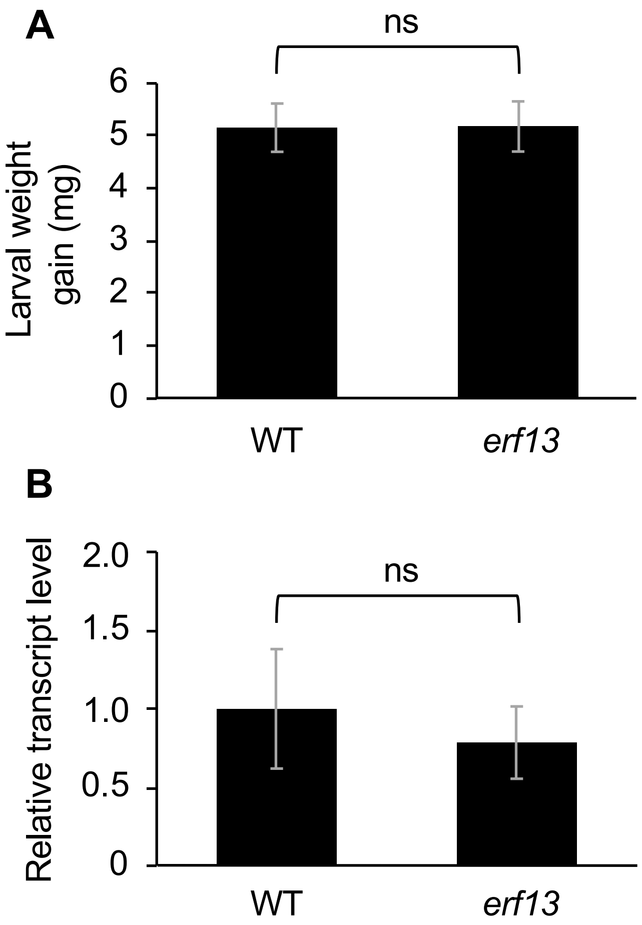
A
ns
Larval weight gain (mg)
6
5
4
3
2
1
0
WT
erf13
B
Relative transcript level
2.0
1.5
1.0
0.5
0
ns
WT
erf13

Figure S2

FIGURE S1. Transcriptional regulation of *CRKs*. Relative transcript levels of *CRK2* and *CRK3* in leaves of transgenic Arabidopsis lines expressing vector control (VC), *CRK2* or *CRK3*. Transcript levels of genes were measured by RT-qPCR and normalized by those of *ACT8*. Data represent the mean and standard error (n = 4-6). Data marked with an asterisk are significantly different from those of VC, based on a one-way ANOVA with Holm's sequential Bonferroni post-hoc test (***, $P < 0.001$).

FIGURE S2. Defense property of wild type (WT) and ERF13 mutant. (A) The net body weight that *Spodoptera litura* larvae gained during 3 days after they had been placed on potted plants of WT (ecotype Landsberg erecta [Ler]) or *erf13* mutant. Data represent the mean and standard error (n = 20-21). **(B)** Transcript levels of *PDF1.2* in the leaves of WT and *erf13* mutant. Transcript levels of genes were measured by RT-qPCR and normalized by those of *ACT8*. Data represent the mean and standard error (n = 4-5). ns, not significant, based on Student's *t*-test ($P > 0.05$).
